# Facilely Fabricating F-Doped Fe_3_N Nanoellipsoids Grown on 3D N-Doped Porous Carbon Framework as a Preeminent Negative Material

**DOI:** 10.3390/molecules29050959

**Published:** 2024-02-22

**Authors:** Dan Zhang, Chunyan Zhang, Huishi Xu, Zhe Huo, Xinyu Shi, Xiaodi Liu, Guangyin Liu, Chuang Yu

**Affiliations:** 1 College of Chemistry and Pharmaceutical Engineering, Nanyang Normal University, Nanyang 473061, China; zhangchunyanny@163.com (C.Z.); x3243536058@163.com (H.X.); imkc62@163.com (Z.H.); sxy021112@163.com (X.S.); liuxiaodiny@126.com (X.L.); liugy13@163.com (G.L.); 2 State Key Laboratory of Advanced Electromagnetic Engineering and Technology, School of Electrical and Electronic Engineering, Huazhong University of Science and Technology, Wuhan 430074, China

**Keywords:** metal nitride, Fe_3_N, N-doped porous carbon framework, negative electrode, lithium-ion batteries, electrochemical performance

## Abstract

Transition metal nitride negative electrode materials with a high capacity and electronic conduction are still troubled by the large volume change in the discharging procedure and the low lithium ion diffusion rate. Synthesizing the composite material of F-doped Fe_3_N and an N-doped porous carbon framework will overcome the foregoing troubles and effectuate a preeminent electrochemical performance. In this study, we created a simple route to obtain the composite of F-doped Fe_3_N nanoellipsoids and a 3D N-doped porous carbon framework under non-ammonia atmosphere conditions. Integrating the F-doped Fe_3_N nanoellipsoids with an N-doped porous carbon framework can immensely repress the problem of volume expansion but also substantially elevate the lithium ion diffusion rate. When utilized as a negative electrode for lithium-ion batteries, this composite bespeaks a stellar operational life and rate capability, releasing a tempting capacity of 574 mAh g^–1^ after 550 cycles at 1.0 A g^–1^. The results of this study will profoundly promote the evolution and application of transition metal nitrides in batteries.

## 1. Introduction

The significant consumption of conventional fossil energy sources can touch off various serious problems, such as rapidly increasing prices, environmental pollution and global warming. All of these problems have forced people to develop alternative energy sources. In recent years, scientists have successively developed wind energy, solar energy, fuel cells, supercapacitors and lithium-ion batteries (LIBs). Among these, LIBs are regarded as the most popular energy sources in people’s daily lives due to their durable operation life, low usage cost and environmental pollution [1,2,3,4]. Nonetheless, the presently utilized graphite negative electrodes for LIBs bespeak an inferior energy density, meaning that they need a long charging time, which profoundly constrains the whirlwind evolution of LIBs employed in electric automobiles [5,6,7]. Thereupon, the study of negative electrode materials with a high capacity plays a crucial role in the speedy development and application of LIBs in electric automobiles.

The transition metal oxides used as negative electrode material have a high capacity, approximately 2–3 times higher than graphite, and have attracted widespread attention [8,9]. Nevertheless, most transition metal oxides display poor conductivity, thereby leading to the low utilization efficiency and rate performance of active materials [10,11]. To improve the low conductivity of transition metal oxide electrode materials, generally, they are combined with high-conductivity carbon materials [12,13]. For such composite materials, transition metal oxides only come into contact with conductive carbon on their surface, and the internal conductivity of the transition metal oxide materials has not been improved. Under low load conditions, the content of transition metal oxide with a high capacity is low, and the final electrochemical performance of the composite material is not ideal. As the load of transition metal oxides increases, the poor conductivity of the transition metal oxide phase leads to a great decrease in their performance rate, which limits their final application in LIBs. Hence, the development of new negative electrode materials with both high conductivity and a high capacity is of great scientific significance and application value for LIBs in electric automobiles.

Transition metal nitrides are classic interstitial metal compounds with high melting points, high thermal conductivity, high density, high hardness and good chemical stability [14,15]. Moreover, the nitrogen atoms in transition metal nitrides occupy gaps in the metal lattices, leading to lattice expansion and an increase in d electrons’ density of states so that transition metal nitrides exhibit excellent conductivity, similar to metals [16,17]. Transition metal nitrides have the characteristics of high capacity, high conductivity and high density, and are expected to become fashionable negative electrode materials for LIBs in electric automobiles. However, the utilization of transition metal nitrides as negative electrode materials for LIBs is still troubled by a large volume change and a low lithium ion diffusion rate [18,19]. Aimed at the foregoing troubles, integrating transition metal nitrides with an N-doped porous carbon framework is perceived as an efficacious solution, which would substantially reduce the volume expansion in the discharging procedure and improve the electrical conductivity of the final composite [20,21,22]. Furthermore, ion doping into transition metal nitrides is certificated to be a valid solution to elevating the intrinsic lithium ion diffusion rate of transition metal nitrides [23,24,25]. Among a variety of elements, F is perceived as a fine anion dopant [26,27]. To sum up, there is an urgent need to prepare the composite material of F-doped transition metal nitrides and an N-doped porous carbon framework, and effectuate its preeminent electrochemical performance. Furthermore, most transition metal nitrides are prepared under ammonia atmosphere conditions, which increase the security risks, environmental pollution and production costs. Thus, preparing transition metal nitrides under non-ammonia atmosphere conditions is also very significant for the people and factories interested in transition metal nitrides.

In this study, we have created a simple route to obtain the composite material of F-doped Fe_3_N nanoellipsoids and a 3D N-doped porous carbon framework (abbreviated to F-Fe_3_N/NPCF) under non-ammonia atmosphere conditions. The F-Fe_3_N/NPCF composite can not only tremendously reduce the problem of volume expansion, but also prominently elevate the lithium ion diffusion rate. When utilized as a negative electrode for LIBs, the F-Fe_3_N/NPCF composite bespeaks a sterling electrochemical performance.

## 2. Results and Discussion

Our synthetic method includes the following two steps: the preparation of the sol, and the calcination process. In the process of sol preparation, if the Fe source used is FeCl_2_, FeCl_3_ or Fe(CH_3_COO)_2_, a uniform gel will not be obtained. In the process of calcination, polyacrylic amide is not only a carbon source but also a nitrogen source, and NH_4_F is a fluorine source and an additional nitrogen source. Sodium chloride is a template for the manufacturing of porous structures. With the development of the steel industry, the amount of liquid waste generated by the surface cleaning of steel is very large. For example, in China, there are millions of cubic meters of liquid waste generated by the surface cleaning of steel every year. This liquid waste causes serious pollution in the environment and is therefore included in the national hazardous waste list. Iron ions are the main metal ions in clean liquid waste on the surface of steel. The preparation of high-value products from clean liquid waste on the surface of steel is of great significance for waste resource utilization and environmental protection. The waste liquid of iron ions can be treated with polyacrylic amide (our main raw material) for aggregation and sedimentation and the cost of polyacrylic amide is very low. Therefore, the implementation of the above research ideas can provide novel solutions for the treatment and recycling of clean liquid waste on the surface of steel.

**Figure 1:**
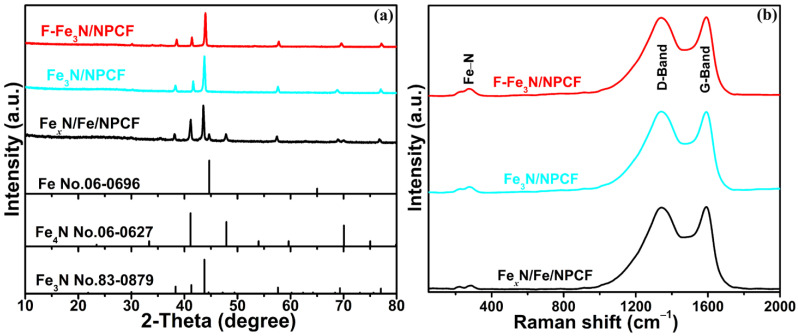
(**a**) The XRD patterns and (**b**) the Raman spectrum for the F-Fe_3_N/NPCF, Fe_3_N/NPCF and Fe*_x_*N/Fe/NPCF composites.

The crystal structures of three prepared samples were explored through XRD. As shown in Figure 1a, all the diffraction peaks of F-Fe_3_N/NPCF and Fe_3_N/NPCF products conform to the classic Fe_3_N phase (JCPDS No. 83-0879). It is worth noting that the XRD peaks of F-Fe_3_N/NPCF present a little deviation to a high angle, proving that F ions with a smaller radius are successfully doped into Fe_3_N to substitute for N ions. For the Fe*_x_*N/Fe/NPCF product, there are additional peaks of Fe_4_N and Fe beyond the main peaks of Fe_3_N. This result implies that an additional nitrogen source is essential for the preparation of pure Fe_3_N. In an effort to investigate the minute structural message in the F-Fe_3_N/NPCF, Fe_3_N/NPCF and Fe*_x_*N/Fe/NPCF samples, the Raman spectrum is exhibited in Figure 1b. The two peaks at 1338–1342 cm^–1^ and 1588–1592 cm^–1^ that emerge in the three samples conform to the D-band and G-band of NPCF [28]. Furthermore, the peaks at 200–300 cm^–1^ of the three samples correspond to the stretching vibration of the Fe-N bond for iron nitrides, thereby consistent with reports in the literature [29,30]. The above result suggests that our products are composites of carbon and iron nitride. The TGA curve (Figure S1, Supplementary Materials) of the F-Fe_3_N/NPCF product could be divided into the following two stages: 22–200 °C and 200–800 °C. Below 200 °C, there is a mass reduction of around 8.9% (wt%), which resulted from the removal of adsorbed molecules, such as H_2_O and CO_2_. For the stage of 200–800 °C, the Fe_3_N phase would be oxidized to Fe_2_O_3_, along with the conversion of carbon to CO_2_ gas. On the basis of the remainder mass of Fe_2_O_3_, we could calculate a mass of 43.2% for Fe_3_N. Therefore, the mass of carbon for the F-Fe_3_N/NPCF product is around 47.9% (1–8.9%–43.2%). An elemental (C, H, N) analysis for NPCF suggests that its nitrogen content is approximately 8.2 wt%.

**Figure 2:**
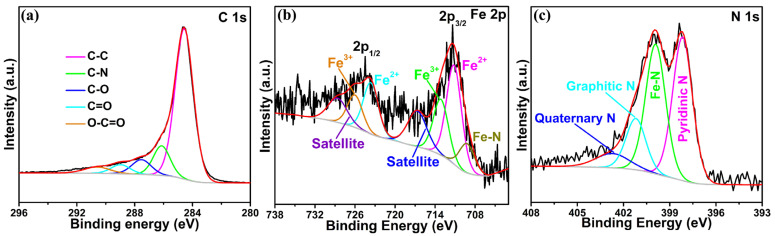
The XPS spectra of the C 1s region (**a**), the Fe 2p region (**b**) and the N 1s region (**c**) of the F-Fe_3_N/NPCF composite.

XPS was employed to ulteriorly derive the chemical state of the F-Fe_3_N/NPCF composite. The narrow-scan XPS spectra of C 1s, Fe 2p and N 1s are exhibited in Figure 2a,c. The C 1s spectrum (Figure 2a) gives five fitted peaks (284.6, 286.1, 287.5, 289.0, 290.6 eV), which tally with the C–C, C–N, C–O, C=O and O–C=O bonds [31], respectively. The appearance of a C–N bond means that the carbon in the F-Fe_3_N/NPCF composite is doped by carbon. The Fe 2p spectrum is displayed in Figure 2b. The two fitted peaks seated at 711.2 and 723.6 eV signify the emergence of an Fe^2+^ state in the composite, whereas the two fitted peaks situated at 713.2 and 726.0 eV belong to an Fe^3+^ state. The two peaks at 716.7 and 728.6 eV correspond to the satellite peaks for 2p_3/2_ and 2p_1/2_. Furthermore, the featured peak at 709.2 eV is in line with the Fe–N bond. These results are in keeping with previous data for typical Fe_3_N materials in the literature [32,33]. The N 1s XPS spectrum (Figure 2c) gives four fitted peaks at 398.2, 399.9, 401.2 and 402.7 eV, which conform to the pyridinic N, Fe–N, graphitic N and quaternary N [34,35], respectively. This result also verifies that the carbon in the F-Fe_3_N/NPCF composite is doped by carbon, and as such is consistent with the result of C 1s. The F 1s spectrum (Figure S2) exhibits one peak at 684.9 eV, which resulted from the Fe–F bond [36]. The above result ulteriorly proves that the F was doped into Fe_3_N. In addition, the F content in the F-Fe_3_N/NPCF composite was estimated to be about 4.3 at% through the XPS analysis.

**Figure 3:**
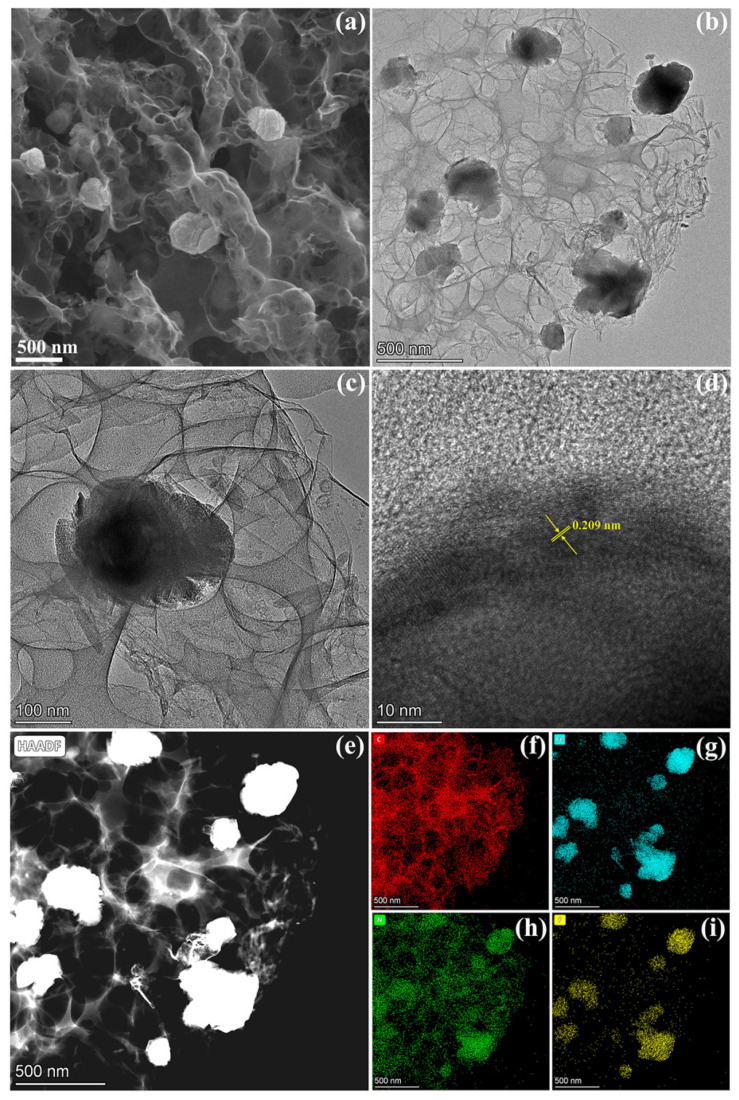
The SEM image (**a**), TEM images (**b**,**c**), HRTEM image (**d**), the corresponding HAADF image of the elemental mapping (**e**) and the EDS maps of the elemental C (**f**), Fe (**g**), N (**h**) and F (**i**) for the F-Fe_3_N/NPCF composite.

Figure 3a gives the SEM image of the F-Fe_3_N/NPCF composite. It is easy to observe that the morphology of most Fe_3_N particles is that of irregular nanoellipsoids, and that they are attached to the 3D N-doped porous carbon framework. The TEM images of the F-Fe_3_N/NPCF composite in Figure 3b,c also confirm that irregular Fe_3_N nanoellipsoids have grown on the 3D N-doped porous carbon framework. Figure 3d presents an HRTEM image of the F-Fe_3_N/NPCF composite. The palpable span of the lattice fringe is 0.209 nm, thereby tallying with the (111) plane for the Fe_3_N. Figure 3e–i show the elemental mapping images of the prepared F-Fe_3_N/NPCF composite. The accumulation of Fe, N and F elements in the position of nanoellipsoids and the uniform C and N elements in position on the 3D N-doped porous carbon framework demonstrates that irregular F-doped Fe_3_N nanoellipsoids can grow on a 3D N-doped porous carbon framework.

**Figure 4:**
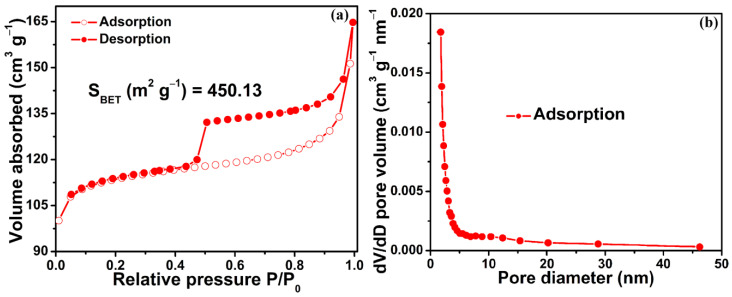
The N_2_ adsorption–desorption isotherm (**a**) and pore size distribution (**b**) of the F-Fe_3_N/NPCF composite.

In order to deeply probe the porous structure of the F-Fe_3_N/NPCF composite, N_2_ adsorption/desorption characterization was utilized. Figure 4a gives the N_2_ sorption isotherm of the F-Fe_3_N/NPCF composite, which displays a type IV pattern. This result implies that the F-Fe_3_N/NPCF composite contains a lot of mesopores [37,38]. The F-Fe_3_N/NPCF composite has a high BET-specific surface area of 450.13 m^2^ g^–1^ and a pore volume of 0.26 cm^3^ g^–1^. Figure 4b shows the pore size distribution of the F-Fe_3_N/NPCF composite. Obviously, the pore sizes of the F-Fe_3_N/NPCF composite are primarily distributed from 2 to 5 nm. The main sources of porous structure for the F-Fe_3_N/NPCF composite are likely to be the decomposition of the polyacrylic amide in the calcination process and the removal of the sodium chloride template in the washing process [39].

**Figure 5:**
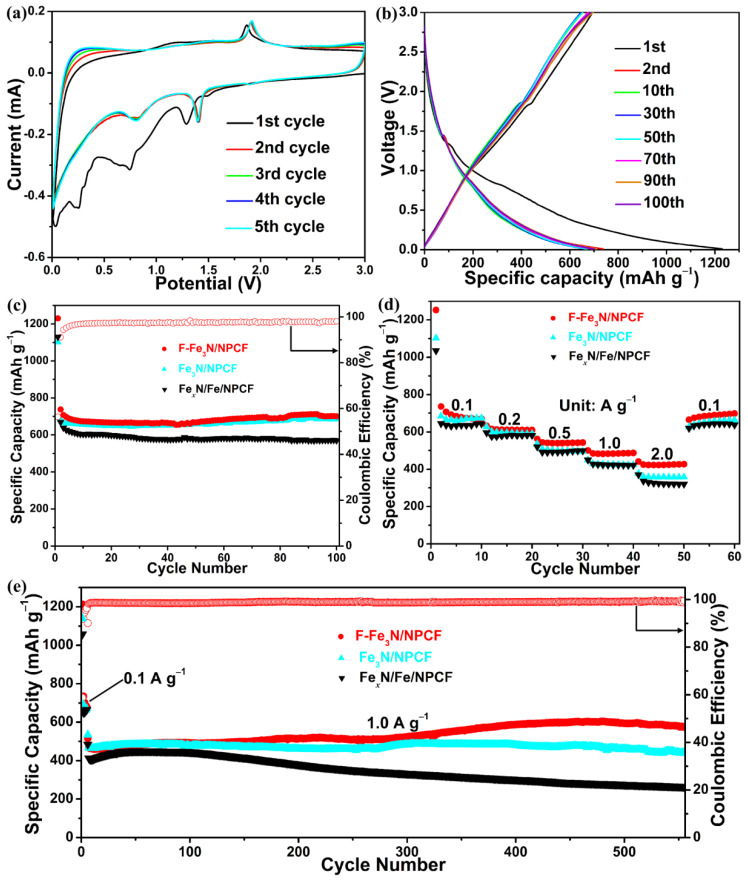
(**a**) The CV curves at a scanning speed of 0.2 mV s^–1^ and (**b**) the discharging–charging potential profiles of the F-Fe_3_N/NPCF negative electrode at 0.1 A g^–1^. (**c**) The cycling performance of the F-Fe_3_N/NPCF, Fe_3_N/NPCF and Fe*_x_*N/Fe/NPCF negative electrodes at 0.1 A g^–1^ and the Coulombic efficiency of the F-Fe_3_N/NPCF negative electrode. (**d**) The rate capability of the F-Fe_3_N/NPCF, Fe_3_N/NPCF and Fe*_x_*N/Fe/NPCF negative electrodes. (**e**) The cycling performance of the F-Fe_3_N/NPCF, Fe_3_N/NPCF and Fe*_x_*N/Fe/NPCF negative electrodes at 1.0 A g^–1^ after activation and the Coulombic efficiency of the F-Fe_3_N/NPCF negative electrode.

The electrochemical performances of F-Fe_3_N/NPCF, Fe_3_N/NPCF and Fe*_x_*N/Fe/NPCF were researched as negative materials utilized in LIBs. Figure 5a shows the initial five cyclic CV curves of the F-Fe_3_N/NPCF negative electrode at a scanning speed of 0.2 mV s^–1^. For the foremost discharging process, there are three distinct reduction peaks in total. The reduction peak located at 1.29 V is likely to be attributed to the embedment of a lithium ion into Fe_3_N and the generation of Li*_x_*Fe_3_N [40]. The reduction peak located at 0.75 V tallies with the transformation of Li*_x_*Fe_3_N to iron elemental and Li_3_N [41]. The peak located at 0.25 V vanishes in the later discharging processes, which could stem from the formation of an SEI film [42]. For the later discharging processes, two reduction peaks shift to a relatively high potential of 1.40 and 0.81 V. In the foremost charging process, the F-Fe_3_N/NPCF negative electrode only gives an evident oxidation peak located at 1.87 V, which corresponds to the release of lithium ion from the Li_3_N and the conversion of iron elemental to Fe_3_N [43]. For the later charging processes, the oxidation peak shifts to a relatively high potential of 1.91 V. Comparing the next four cyclic curves, it is uncomplicated to find that they have barely changed, thus validating the salient electrochemical reversibility of the F-Fe_3_N/NPCF negative electrode.

Figure 5b offers diverse discharging–charging potential profiles from 0.01 to 3 V for the F-Fe_3_N/NPCF negative electrode at 0.1 A g^–1^. The foremost discharge and charge capacities of the F-Fe_3_N/NPCF negative electrode are 1230 and 694 mAh g^–1^, and its foremost Coulombic efficiency is 56%. The capacity loss of the F-Fe_3_N/NPCF negative electrode for the foremost cycle is likely attributed to the yield of the SEI layer. After the 1st cycle, the alterations for the potential curves are terribly tiny, thereby implying that the F-Fe_3_N/NPCF negative electrode has stellar electrochemical reversibility.

Figure 5c shows the cycling performances of the F-Fe_3_N/NPCF, Fe_3_N/NPCF and Fe*_x_*N/Fe/NPCF negative electrodes at 0.1 A g^–1^ for one hundred cycles. After one hundred cycles, the F-Fe_3_N/NPCF negative electrode still shows a nice discharge capacity of 701 mAh g^–1^, which is 95% of the value for the 2nd discharge capacity. The mean Coulombic efficiency from four to one hundred laps is roughly 98%. These results imply the fine electrochemical fixity of the F-Fe_3_N/NPCF negative electrode. Notably, there is a small increase in capacity during the cycling process. Based on previous reports, the increase in capacity during the cycling process can be attributed to the pulverization of particles during this process, along with the decreased particle sizes and the newly formed electrochemical active sites [44]. Meanwhile, the Fe_3_N/NPCF negative electrode merely supplies a comparatively smaller discharge capacity of 686 mAh g^–1^ after one hundred laps. By contrast, the Fe*_x_*N/Fe/NPCF negative electrode just shows the lowest discharge capacities of 570 mAh g^–1^ after one hundred laps.

Figure 5d shows the discharge capacities for diverse current densities between 0.1 and 2.0 A g^–1^. It is apparent that the F-Fe_3_N/NPCF negative electrode bespeaks the best rate capability. It shows discharge capacities of 670, 610, 542, 487 and 427 mAh g^–1^ at each 10th cycle when the current density is increased from 0.1 to 2.0 A g^–1^. Even when the current density returns to 0.1 A g^–1^, the discharge capacity goes back to 698 mAh g^–1^, as before. The discharge capacities of the Fe_3_N/NPCF negative electrode are smaller than the F-Fe_3_N/NPCF negative electrode and are merely 590, 495, 424 and 357 mAh g^–1^ at each 10th cycle at 0.2, 0.5, 1.0 and 2.0 A g^–1^. Compared with the F-Fe_3_N/NPCF negative electrode, the Fe*_x_*N/Fe/NPCF negative electrode also bespeaks poorer discharge capacities of 581, 498, 422 and 320 mAh g^–1^ at each 10th cycle at 0.2, 0.5, 1.0 and 2.0 A g^–1^. Figure 5e provides the cycling performances of the F-Fe_3_N/NPCF, Fe_3_N/NPCF and Fe*_x_*N/Fe/NPCF negative electrodes at 1.0 A g^–1^ for 550 cycles after five cycles of activation at 0.1 A g^–1^. From this graph, it can be determined that the F-Fe_3_N/NPCF negative electrode also carries a preeminent cyclic lifespan. It offers the highest discharge capacity of 574 mAh g^–1^ after 550 cycles at 1.0 A g^–1^ and bespeaks a mean Coulombic efficiency of 99% from the first cycle to 550th cycle. As comparisons, the Fe_3_N/NPCF and Fe*_x_*N/Fe/NPCF negative electrodes only deliver lower discharge capacities of 443 and 265 mAh g^–1^ after 550 cycles at 1.0 A g^–1^.

**Figure 6:**
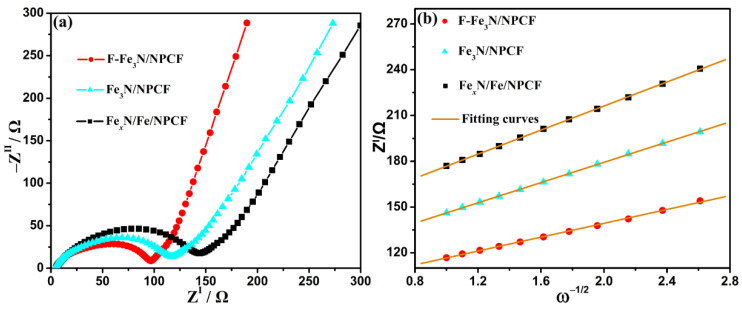
(**a**) The electrochemical impedance spectra after 5 cycles at 0.1 A g^–1^ and (**b**) data fitting results of plots for the F-Fe_3_N/NPCF, Fe_3_N/NPCF and Fe*_x_*N/Fe/NPCF negative electrodes.

The electrochemical impedance spectra (EIS) of the F-Fe_3_N/NPCF, Fe_3_N/NPCF and Fe*_x_*N/Fe/NPCF negative electrodes after 5 cycles are shown in Figure 6a. They are fitted via the equivalent circuit in Figure S3 and all fitted data are offered in Table S1. The SEI film resistances (R_f_) for the F-Fe_3_N/NPCF, Fe_3_N/NPCF and Fe*_x_*N/Fe/NPCF negative electrodes are 34, 50 and 65 Ω, and the charge transfer resistances (R_ct_) are 65, 74 and 86 Ω. The lower R_f_ and R_ct_ values of the F-Fe_3_N/NPCF negative electrode would contribute to the speedy lithium ion and electron movement [45,46]. Furthermore, we counted three diffusion coefficients of lithium ion (D_Li_^+^) for the F-Fe_3_N/NPCF, Fe_3_N/NPCF and Fe*_x_*N/Fe/NPCF negative electrodes abiding by Equation (1) [47]:

D_Li_^+^ = (R^2^T^2^)/(2A^2^n^4^F^4^ C^2^*σ*^2^) (1)

In Equation (1), R, T, A, n, F and C represent the gas constant, Kelvin temperature, surface area of the electrode, electron number in the electrochemical reaction, Faraday constant and concentration of lithium ion in the electrode, respectively. The value of the gradient fitted from the Z^I^–*ω*^–1/2^ data in the Warburg range is *σ*. The *σ* values of F-the Fe_3_N/NPCF, Fe_3_N/NPCF and Fe*_x_*N/Fe/NPCF negative electrodes are 22.7, 33.2 and 39.4, as exhibited in Figure 6b. According to Equation (1), the counted D_Li_^+^ of the Fe_3_N@C/NPCN negative electrode is 1.9 × 10^–15^ cm^2^ s^–1^, which is far bigger than the values of the Fe_3_N/NPCF and Fe*_x_*N/Fe/NPCF negative electrodes (9.0 × 10^–16^ cm^2^ s^–1^ and 6.4 × 10^–16^ cm^2^ s^–1^). Consequently, the F-Fe_3_N/NPCF negative electrode bespeaks an optimal electrochemical performance.

**Figure 7:**
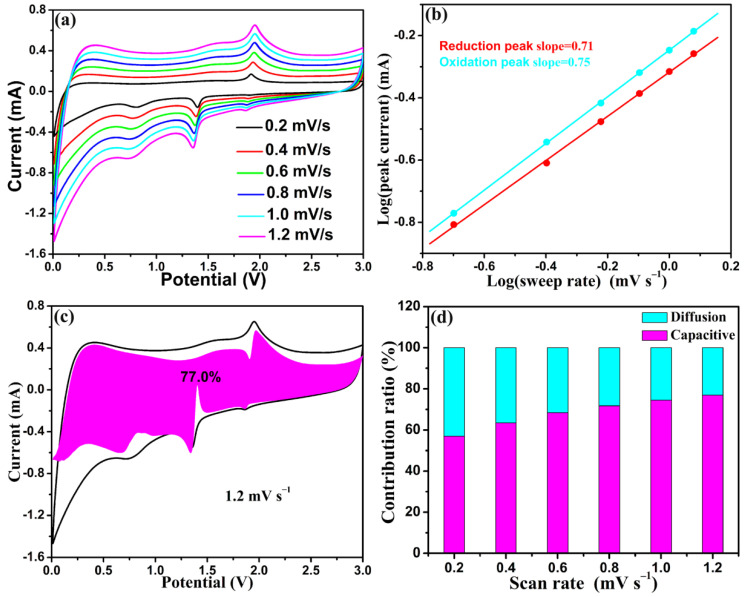
CV curves at diverse sweep rates (**a**), log (peak current) versus log (sweep rate) chart at specific current peaks (**b**), CV outline of the capacitive contribution at 1.2 mV s^–1^ (**c**) and normalized diffusion and capacitive ratios at diverse sweep rates (**d**) for the F-Fe_3_N/NPCF negative electrode.

CV curves with diverse scanning rates from 0.2 to 1.2 mV s^–1^ were created for ulteriorly studying the reason for the good electrochemical performance of the F-Fe_3_N/NPCF negative electrode. From Figure 7a, we can see that the changes in the configuration and sites of the anodic and cathodic peaks for the F-Fe_3_N/NPCF negative electrode are relatively small if the scanning rate is altered. This result demonstrates that the F-Fe_3_N/NPCF negative electrode has speedy electrochemical reaction kinetics [48]. More often than not, the current density (*i*) and scanning speeds (*v*) abide by Equation (2) [49]:

*i* = a*v*^b^ (2)

In light of Equation (2), we can find that the value of the gradient for the log *v*–log *i* data is b. 0.5 < b < 1, signaling a hybrid procedure consisting of a capacitive-dominated procedure and an ionic diffusion-controlled procedure [50]. As shown in Figure 7b, the b value of the cathodic peak is 0.71 and the anodic peak is 0.75, respectively, thereby proving that the storage mechanism of the lithium ion for the F-Fe_3_N/NPCF negative electrode is maneuvered with the coinstantaneous capacitive and ionic diffusion-controlled procedures. Added to that, the ratios of the capacitive-governed procedure and the ionic diffusion-governed procedure could be attained via Equation (3) [51]:

*i* = k_1_*v* + k_2_*v*^1/2^ (3)

Figure 7c,d shows that the ratios for the capacitive-governed procedure with the F-Fe_3_N/NPCF negative electrode are 57%, 63.4%, 78.4%, 71.7%, 74.6% and 77.0% when the scanning rates are altered from 0.2 to 1.2 mV s^–1^. The foregoing values certify that the capacitive-governed procedure possesses a bigger ratio for the F-Fe_3_N/NPCF negative electrode at each scanning rate, particularly at speedy scanning rates. A capacitive-governed procedure will help the speedy storage of lithium ion, and enhance the attractive rate performance of the F-Fe_3_N/NPCF negative electrode [52].

Table 1 contrasts the electrochemical performances of the F-Fe_3_N/NPCF negative electrode with the results in the literature on transition metal nitride negative electrodes. It is apparent that the F-Fe_3_N/NPCF negative electrode exhibits better cycling stability and rate performance than all of them, including Fe_3_N@C [53], Fe_3_N/rGO [54], FeN/PC [55], Fe_2_N@AC@rGO [35], Fe_2_N@CNFs [56], Fe_2_N/rGO [42], Fe_2_N@Fe_3_O_4_/VN [57], GQD@hNi_3_N [58], (Ni/Co)_3_N MC@HC [20], Co-VN@C [59] and MoS_2_/MoN [60]. The good cycling stability and outstanding rate performance of the F-Fe_3_N/NPCF negative electrode may be due to its peculiar structure. The Fe_3_N nanoellipsoids combined with the 3D N-doped porous carbon framework markedly restrain the volume expansion in the discharging procedure. In addition, the doping of F promotes the lithium ion diffusion rate.

## 3. Experimental Section

### 3.1. Synthesis of F-Fe_3_N/NPCF Composite

An F-Fe_3_N/NPCF sample was prepared according to the following procedures. To begin with, 1.5 mmol of sodium ferric ethylenediaminetetraacetate was added to 10 mL of distilled water and stirred for 10 min to obtain solution 1. Later, 0.4 g of polyacrylic amide, 0.1 g of NH_4_F and 1.0 g of NaCl were added to 20 mL of distilled water and stirred for 10 min to obtain solution 2. Then, we dropped solution 1 into solution 2, and stirred for 60 min. The gel formed was placed in a refrigerator at –18 °C for 12 h, and then freeze-dried for 24 h. The resulting powder was heated at 700 °C for 90 min in an inert gas atmosphere. Then, the obtained material was washed with distilled water. The F-Fe_3_N/NPCF sample was collected through vacuum filtration and drying at 60 °C. When the NH_4_F is replaced by urea and the other synthetic conditions remain unchanged, an Fe_3_N/NPCF comparison sample is obtained. When there is no NH_4_F present and the other synthetic conditions remain unchanged, an Fe*_x_*N/Fe/NPCF comparison sample is obtained.

### 3.2. Materials Characterization

The powder X-ray diffraction (XRD) data were obtained with a Rigaku SmartLab SE diffractometer (Rigaku, Tokyo, Japan). The scanning electron microscopic (SEM) images were acquired with a ZEISS GeminiSEM 300 (ZEISS, Oberkochen, Germany). The transmission electron microscopy (TEM) images and the EDS result were obtained with a Thermo Talos F200X G2 (Thermo, Waltham, MA, USA). The nitrogen adsorption isotherms of the samples were measured with a Micromeritics ASAP 2460 (Micromeritics, Norcross, GA, USA). The Raman spectrum was obtained with a Horiba LabRAM HR Evolution (Horiba, Kyoto, Japan). X-ray photoelectron spectroscopy (XPS) was performed with a Thermo Scientific K-Alpha instrument (Thermo Scientific, Waltham, MA, USA). The electrochemical measurements were taken with a Neware Battery Test System and an electrochemical workstation (CHI660C). Further information about the electrode preparation, battery assembly and electrochemical tests is given in the Supporting Information. 

## 4. Conclusions

In conclusion, the composite of F-doped Fe_3_N nanoellipsoids and a 3D N-doped porous carbon framework has been successfully obtained via a simple method under non-ammonia atmosphere conditions. The battery performance measurements certify that the F-Fe_3_N/NPCF negative electrode has an appealing operational life and a transcendent rate capability. Even after 550 cycles at 1.0 A g^–1^, the F-Fe_3_N/NPCF negative electrode still yields a fine capacity of 574 mAh g^–1^. This eminent electrochemical performance may be due to the F-doped Fe_3_N nanoellipsoids being combined with a 3D N-doped porous carbon framework, which profoundly retards the volume expansion but also tremendously increases the lithium ion diffusion rate. Hence, this study could dramatically propel the future application of transition metal nitrides for batteries.

## Figures and Tables

**Table 1:** Electrochemical performances of the newly studied transition metal nitride negative electrodes.

Negative Electrode	First Discharge–Charge Capacity (mA h g^–1^)	Cyclic Performance	Rate Performance
F-Fe_3_N/NPCF (This work)	1230/694 at 0.1 A g^–1^	701 (100 cycles, 0.1 A g^–1^) 574 (550 cycles, 1.0 A g^–1^)	487 (1.0 A g^–1^) 427 (2.0 A g^–1^)
Fe_3_N@C [53]	676/548 at 0.1 A g^–1^	370 (500 cycles, 0.1 A g^–1^)	244 (1.0 A g^–1^)
Fe_3_N/rGO [54]		615 (200 cycles, 0.2 A g^–1^) 513 (200 cycles, 0.5 A g^–1^)	308 (1.0 A g^–1^) 261 (2.0 A g^–1^)
FeN/PC [55]	1081/607 at 0.1 A g^–1^	558 (80 cycles, 0.1 A g^–1^) 420 (250 cycles, 1.0 A g^–1^)	408 (1.0 A g^–1^) 356 (2.0 A g^–1^)
Fe_2_N@AC@rGO [35]		505 (500 cycles, 0.5 A g^–1^)	446 (1.0 A g^–1^) 406 (2.0 A g^–1^)
Fe_2_N@CNFs [56]	900/587 at 0.1 A g^–1^	551 (60 cycles, 0.1 A g^–1^)	392 (1.0 A g^–1^) 343 (2.0 A g^–1^)
Fe_2_N/rGO [42]	1256/804 at 0.1 A g^–1^	578 (500 cycles, 0.1 A g^–1^)	380 (1.0 A g^–1^) 318 (2.0 A g^–1^)
Fe_2_N@Fe_3_O_4_/VN [57]		421 (600 cycles, 1.0 A g^–1^)	244 (1.0 A g^–1^) 212 (2.0 A g^–1^)
GQD@hNi_3_N [58]	1097/550 at 0.4 A g^–1^	450 (500 cycles, 0.4 A g^–1^)	326 (0.8 A g^–1^) 268 (1.6 A g^–1^)
(Ni/Co)_3_N MC@HC [20]		440 (130 cycles, 0.2 A g^–1^) 170 (130 cycles, 0.5 A g^–1^)	
Co-VN@C [59]	830/439 at 0.2 A g^–1^	336 (500 cycles, 0.5 A g^–1^) 324 (200 cycles, 1.0 A g^–1^)	321 (1.0 A g^–1^)
MoS_2_/MoN [60]	820/671 at 0.1 A g^–1^	475 (100 cycles, 0.1 A g^–1^)	345 (2.0 A g^–1^)

## Data Availability

The data presented in this study are available on request from the corresponding author. The data are not publicly available due to institutional restriction.

## Supplementary Materials

The following supporting information can be downloaded at: https://www.mdpi.com/article/10.3390/molecules29050959/s1, Figure S1: TG curve of F-Fe_3_N/NPCF composite; Figure S2: XPS spectrum of the F 1s region for F-Fe_3_N/NPCF composite; Figure S3: Equivalent circuit; Table S1: Electrochemical impedance parameters of the F-Fe_3_N/NPCF, Fe_3_N/NPCF and Fe*_x_*N/Fe/NPCF electrodes obtained from equivalent circuit fitting of experimental data.
