# Supplementary material for: Facilely Fabricating F-Doped Fe3N Nanoellipsoids Grown on 3D N-Doped Porous Carbon Framework as a Preeminent Negative Material
Source: Molecules. 2024 Feb 22;29(5):959. doi: 10.3390/molecules29050959 (PMC10934314; doi:10.3390/molecules29050959)
Supplement: Supplementary file 1 [file molecules-29-00959-s001.zip › molecules-2863834-supplementary.pdf]

# Facilely Fabricating F-Doped $Fe_3N$ Nanoellipsoids Grown on 3D N-Doped Porous Carbon Framework as a Preeminent Negative Material

**Dan Zhang [1,*], Chunyan Zhang [1], Huishi Xu[1], Zhe Huo[1], Xinyu Shi[1], Xiaodi Liu[1], Guangyin Liu[1] and Chuang Yu[2,*]**

[1] College of Chemistry and Pharmaceutical Engineering, Nanyang Normal University, Nanyang 473061, PR China; zhangchunyanny@163.com (C.Z.); x3243536058@163.com (H.X.); imkc62@163.com (Z.H.); sxy021112@163.com (X.S.); liuxiaodiny@126.com (X.L.); liugy13@163.com (G.L.)

[2] State Key Laboratory of Advanced Electromagnetic Engineering and Technology, School of Electrical and Electronic Engineering, Huazhong University of Science and Technology, Wuhan 430074, PR China

* Correspondence: danzhangny@163.com (D.Z.); cyu2020@hust.edu.cn (C.Y.)

**Electrochemical measurements**

The electrochemical performance of the samples was tested using CR2025-type coin cell. The test electrodes were prepared by mixing the samples, carbon black, and binder (polyvinylidene fluoride (PVDF)) at a weight ratio of 7:2:1 in N-methyl-2-pyrrolidinone (NMP). The resulting slurries were coated onto a copper foil and then dried under vacuum at 80 $^oC$ for 12 h. Coin cells were assembled in an argon-filled glovebox ($H_2O$ and $O_2$ < 0.1 ppm) using lithium foil as the counter and reference electrode, a polymer separator (Celgard 2500), and 1 M $LiPF_6$ in EC:DMC:DEC (1:1:1 in volume) as the electrolyte. The cells were tested using a LANHE Battery Test System in the potential range between 0.01 and 3 V at room temperature. Cyclic voltammetry (CV) was measured using an electrochemical workstation (CHI660C). Electrochemical impedance spectra (EIS) were characterized by the same instrument over a frequency range of 100 kHz to 0.01 Hz.

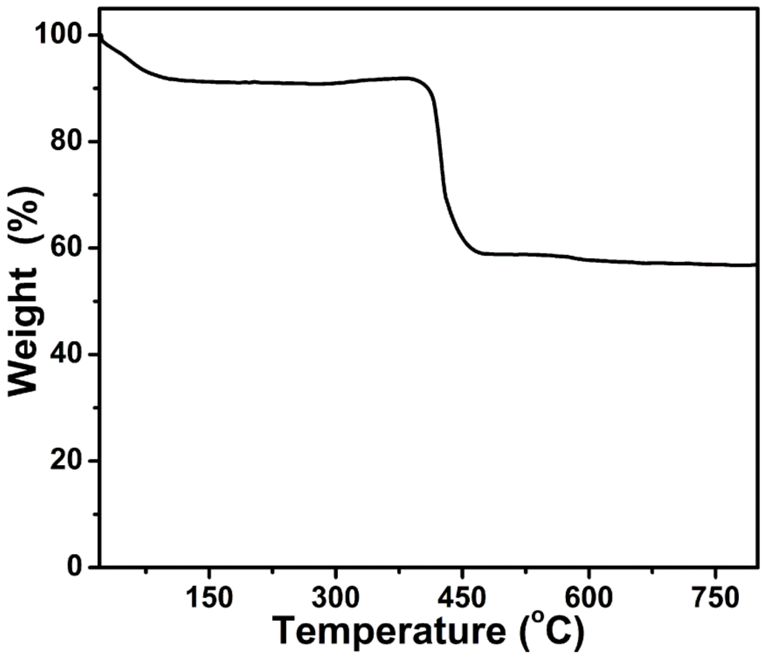


**Figure S1** TG curve of F-$Fe_3N$/NPCF composite.

TGA curve for F-$Fe_3N$/NPCF composite can be divided into two parts: from 20 °C to 200 °C and from 200 °C to 800 °C. In the first part, a mass loss of approximately 8.9% (wt %) is observed below 200 °C on the TG curve, which can be attributed to the desorption of adsorbed molecules (e.g., $CO_2$, $H_2O$). In the second part, from 200 °C to 800 °C, the $Fe_3N$ phase was oxidized into $Fe_2O_3$, accompanying the complete removal of carbon. Based on the remaining weight percent of $Fe_2O_3$, we can calculate the weight percent of 43.2% for $Fe_3N$. Thus, the carbon content for the F-$Fe_3N$/NPCF composite is approximately 47.9% (1-8.9%-43.2%).

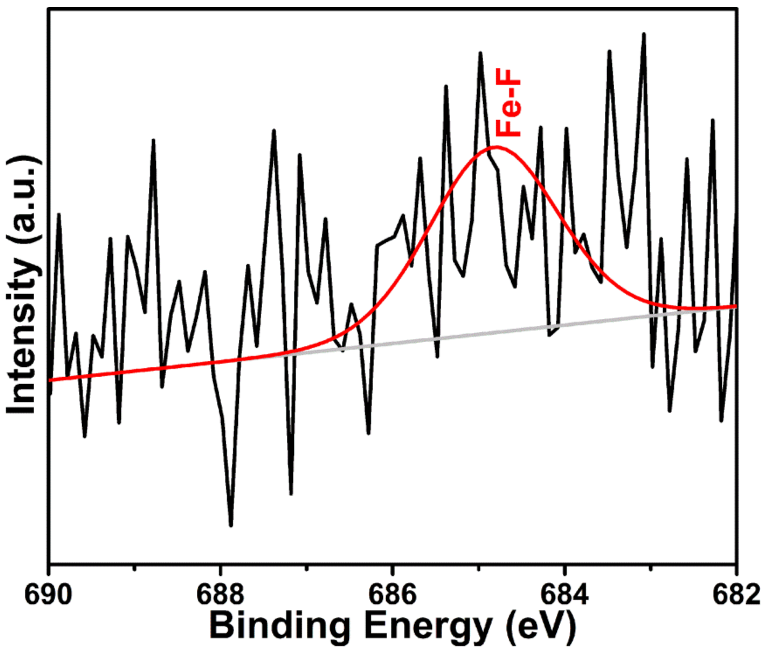


**Figure S2** XPS spectrum of the F 1s region for F-$Fe_3N$/NPCF composite.

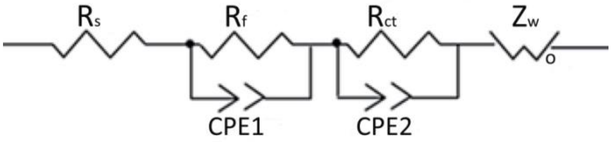


**Figure S3** Equivalent circuit.

**Table S1** Electrochemical impedance parameters of the F-$Fe_3N$/NPCF, $Fe_3N$/NPCF and $Fe_xN$/Fe/NPCF electrodes obtained from equivalent circuit fitting of experimental data.

| Sample | Cycle number | $R_s$/Ohm | $R_f$/Ohm | $R_{ct}$/Ohm |
|---|---|---|---|---|
| F-$Fe_3N$/NPCF | after five cycles | 4 | 34 | 65 |
| $Fe_3N$/NPCF | after five cycles | 4 | 50 | 74 |
| $Fe_xN$/Fe/NPCF | after five cycles | 4 | 65 | 86 |
